# Selection and performance of village health teams (VHTs) in Uganda: lessons from the natural helper model of health promotion

**DOI:** 10.1186/s12960-015-0074-7

**Published:** 2015-09-07

**Authors:** Emmanueil Benon Turinawe, Jude T. Rwemisisi, Laban K. Musinguzi, Marije de Groot, Denis Muhangi, Daniel H. de Vries, David K. Mafigiri, Robert Pool

**Affiliations:** University of Amsterdam, Amsterdam, Netherlands; Makerere University, Kampala, Uganda

**Keywords:** Community health workers, Village health teams, Natural helpers

## Abstract

**Background:**

Community health worker (CHW) programmes have received much attention since the 1978 Declaration of Alma-Ata, with many initiatives established in developing countries. However, CHW programmes often suffer high attrition once the initial enthusiasm of volunteers wanes. In 2002, Uganda began implementing a national CHW programme called the village health teams (VHTs), but their performance has been poor in many communities. It is argued that poor community involvement in the selection of the CHWs affects their embeddedness in communities and success. The question of how selection can be implemented creatively to sustain CHW programmes has not been sufficiently explored. In this paper, our aim was to examine the process of the introduction of the VHT strategy in one rural community, including the selection of VHT members and how these processes may have influenced their work in relation to the ideals of the natural helper model of health promotion.

**Methods:**

As part of a broader research project, an ethnographic study was carried out in Luwero district. Data collection involved participant observation, 12 focus group discussions (FGDs), 14 in-depth interviews with community members and members of the VHTs and four key informant interviews. Interviews and FGD were recorded, transcribed and coded in NVivo. Emerging themes were further explored and developed using text query searches. Interpretations were confirmed by comparison with findings of other team members.

**Results:**

The VHT selection process created distrust, damaging the programme’s legitimacy. While the Luwero community initially had high expectations of the programme, local leaders selected VHTs in a way that sidelined the majority of the community’s members. Community members questioned the credentials of those who were selected, not seeing the VHTs as those to whom they would go to for help and support. Resentment grew, and as a result, the ways in which the VHTs operated alienated them further from the community. Without the support of the community, the VHTs soon lost morale and stopped their work.

**Conclusion:**

As the natural helper model recommends, in order for CHW programmes to gain and maintain community support, it is necessary to utilize naturally existing informal helping networks by drawing on volunteers already trusted by the people being served. That way, the community will be more inclined to trust the advice of volunteers and offer them support in return, increasing the likelihood of the sustainability of their service in the community.

## Introduction

In 1978, world leaders created the Declaration of Alma-Ata, which reaffirmed access to health as a fundamental human right and identified primary health care as the key to the attainment of the goal of health for all. The 30th anniversary of Alma-Ata coincided with the halfway mark of the United Nations’ Millennium Development Goals, stimulating discussion about the role of primary health care in facilitating the achievement of those goals and led to revitalized calls for use of community health workers (CHWs) as a form of community participation [1]. CHWs help individuals and groups in their own communities access health and social services and educate them about various health issues [2]. Many studies have documented the advantages of CHW programmes [3-7], including that such programmes can enhance community participation [8,9].

Initially, international health actors promoted CHWs as a means to achieve the World Health Organization’s goal of health access for all by the year 2000 through social interventions for behaviour change [10,11]. Optimism about the potential of CHWs led to the increased desirability of community-based health interventions [12]. As early as 2000, however, the optimism about CHW programmes began to fade, as there was little progress towards the achievement of health goals for the poor, and CHW programmes showed heterogeneous outcomes [2,13]. Further, CHW programmes have been characterized by high levels of attrition through resignations, terminations and relocations [14,15].

Financial compensation for CHWs has been, and remains, a contentious issue, especially in poor countries where a large number of CHWs are needed [16,17]. While CHWs are ideally volunteers, in practice, many programmes have financially rewarded CHWs, even hiring them as salaried assistants [18,19]. It has been argued that financial incentives reduce volunteers’ willingness to work without pay [7] and that such incentives are likely to be insufficient, leading to high attrition [20,21]. Despite these issues, both international and national stakeholders have continued to reaffirm the importance of CHWs, and many countries have implemented CHW programmes at the national level, some with remuneration [22-24]. The calls by local and international stakeholders for expanding the use of CHWs by 2015 have led to questions about relying on volunteers to deliver services in poor communities, with some arguing for greater creativity in the selection, recruitment and retention of CHWs [25-28].

Though there is consensus that local communities should be involved in the selection of CHWs, questions have remained on how that selection should be structured. A review of studies on CHW programmes noted that authors state that CHW were “selected by the community” without showing how this was done [29,30]. This is problematic if large-scale programmes involving volunteer CHWs are to be sustained in communities [29]. The question that needs to be constantly asked is what is the best way to draw volunteers from a community, without relying on financial incentives?

### The natural helper model: improving the selection of community health workers

The natural helper model (NHM) is based on a simple premise: within every community, an informal helping network already exists. People with problems naturally seek out other people they trust, and interactions are often spontaneous [31]. The NHM taps into and uses this already existing network to disseminate accurate information on health and other social services to the community; since many people are linked to different helping networks simultaneously [32], the dissemination of health messages can be reinforced. According to the NHM, in order to recruit the most suitable candidates for volunteering in the community, all community members’ networks of informal social support should be studied. In this way, the most trusted and motivated helpers, referred to as “natural helpers”, can be identified [33-35]. Natural helpers are then trained to help others more effectively, with the aim of ensuring that a trained natural helper can act as a representative for each of the networks within a community.

Studies conducted between 1945 and 1959 among ethnic communities in South Africa uncovered the structure and function of community helping systems and drew attention to the significance of social networks in community health education [36]. The NHM originated out of these insights and was designed to enhance the ability of individuals to help others through their own existing personal social networks [37,38]. The NHM promotes the utilization of “key persons” within social networks, individuals to whom others “naturally turn for advice emotional support and tangible aid” [39,40]. These persons are respected and trusted, and have a reputation for being good listeners, responsive to the needs of others, and in control of their own life circumstances [41,42]. Utilizing existing community structures increases both the short- and long-term viability of health programmes and their ability to address community needs [38]. While the NHM has been critiqued for its long and rigorous process in identifying natural helpers, others have argued that, for programmes where sustainability is a priority, the trade-off in time and resources during the identification process for natural helpers becomes worthwhile [38,41].

In some instances, natural helpers have been referred to as “lay health advisers”, especially in programmes in the United States focused on minority communities [5]. While lay health advisers are paid workers, natural helpers are not working for any agency but contribute to the community through their own social networks. The two therefore lie at opposite ends of the same continuum [43] of lay community workers. While natural helpers operate within their own social networks, lay health advisers provide support to individuals who may be strangers [44].

### The history and context of village health teams in Uganda

Since the Alma-Ata declaration, successive Ugandan governments have acknowledged the relationship between health and poverty, but unfortunately, political turmoil made interventions impossible until the 1990s, when fragmented community-based interventions by development partners began to be implemented [45]. The 1999 national health policy included community empowerment and mobilization for health as key elements of the national minimum health care package. A programme designed to improve the home-based management of fevers, implemented after the Abuja Declaration of 2000, demonstrated the benefits of community-based interventions and opened up the way for a strategy based on village health teams (VHTs) [46,47].

The selection of VHTs followed a process of building consensus in the community. First, during face-to-face sensitization sessions, community members were educated about the programme and its need for volunteers. The meeting’s facilitator, often a technical person from the district’s health team or the nearest health centre, described the kind of people best suited for selection as VHTs. After sensitization and consensus building among all stakeholders and all households in the village have occurred, a popular vote is held. According to Uganda’s Ministry of Health guidelines, to be selected as a VHT member, a person must meet several criteria: he or she must be above 18 years of age, a village resident, able to read and write in the local language, a good community mobilizer and communicator, a dependable and trustworthy person, someone interested in health and development and someone willing to work for the community. Preference is given to people already serving as CHWs especially if they have served well [48].

Nationally, VHTs are expected to carry out general tasks in all PHC core areas which include home visiting, mobilization of communities for utilization of health services, health promotion and education, management of common illnesses, follow-up of pregnant mothers and newborns, follow-up of discharged patients and those on long-term treatment and community information management [49]. This necessitated generalist training on a range of subjects including interpersonal communication, community mobilization and empowerment, child growth and development, control of communicable diseases, sexual and reproductive health, environmental health, mental health and monitoring record keeping [50]. The target was for all villages to have trained VHTs by 2010, but only 77% of all the districts had achieved this by 2009 [49]. Due to the financial constraints at various districts, recruitment and training of VHTs has been supported by international development partners [51,52]. In Luwero, the implementation of the VHT strategy was supported by the African Medical Research Foundation (AMREF), under its malaria, HIVAIDS, and TB projects. In VHT training, these three diseases received extra emphasis to reflect the interests of AMREF [53]. By June 2011, Luwero district had a functional VHT structure, and the activities of the VHTs were facilitated directly by AMREF. In 2012, however, the project under which the VHTs were supported ended, which left the local government in charge of facilitating the VHTs [54].

By the time fieldwork for this study commenced in August 2012, the activities of the VHTs had ground to a halt following the termination of the AMREF project. Early on, our research team found that motivation had declined among the VHTs due to the departure of AMREF, even though they had been recruited as volunteers and should have been able to work without AMREF. We thus developed an interest in the processes of VHT strategy implementation in Luwero. In this paper, our aim is to examine the introduction of the VHT strategy in the community and the selection and recruitment of VHT members and how this process may have influenced their work in relation to the ideals of the natural helper model of health promotion.

## Setting and methods

Luwero is an ethnically mixed district in central Uganda. Luwero sub-county’s major ethnic group is the Baganda and the dominant language is Luganda. Typical of many rural communities in central Uganda, the main sources of livelihood are peasant agriculture and petty trade in agricultural and household items, sold in food stalls and shops spread around the villages and along the arterial highway connecting Kampala with northern Uganda [55]. Luwero sub-county has a population of 29 904 [56] and is served by a government health centre situated in Kasana town. Like most rural communities in Uganda, the population of Luwero is poor and has limited access to basic health care.

Fieldwork was carried out as part of a broader project called “Developing Sustaining Community Health Resources” (CoHeRe) between July 2012 and April 2014. Data were collected through participant observation, focus group discussions (FGDs) and in-depth interviews. Participant observation provided a point of entry into the community through joining in the activities of daily life, such as community meetings, prayers, weddings and burials. Spontaneous interactions yielded insights into the lives of community members thus facilitating recruitment for other techniques of data collection. Field notes were taken on a daily basis to keep track with common activities. In addition, 18 in-depth interviews were carried out with community members, members of VHTs and other key informants in the local government and leaders of AMREF. Each interview took about 1 h. Twelve FGDs were conducted with community members from different population categories in order to observe convergence of ideas on issues related to the topic of VHTs. These were organized in well-secluded areas to avoid noise. Each group consisted of 6–10 members and lasted an average of 1.5 h. All interviews participants were purposively selected, targeting those willing and with greater understanding of the issues relating to VHTs.

### Ethics considerations

Tape-recording was done after seeking and obtaining permission from the participants. All audio recordings were transcribed into English and stored on password-protected files accessed only by the research group. Pseudonyms are used in the writing of this paper in order to hide the identity of respondents. This study was approved by the University of Amsterdam, Social Science Ethical Advisory Board. In Uganda, ethical clearance was granted by the Institutional Review Board of Makerere University College of Health Sciences and the National Council of Science and Technology.

### Data analysis

Inductive data analysis was guided by three broad tasks: data reduction, data display and conclusion drawing or verification [57]. All interviews and FGDs were conducted in Luganda, recorded and transcribed in English. The transcription was carried out through an iterative process of back and forth reflections on the data to gain “immersion in the details and specifics of the data” and discover “important patterns, themes, and interrelationships” which were then followed up in subsequent interviews [58]. All transcripts were imported into Nvivo10 software for coding and analysis. In addition, text searches were carried out for relevant key words and new emerging themes. The sources were re-read and coded until saturation of themes was achieved. After comparisons with findings from other team members, interpretations were confirmed in the following structure: (a) introduction of and reactions to the VHT programme, (b) VHT selection, (c) community disappointment with and resentment of the VHTs, (d) VHT adaptation in response to community resentment and (e) VHT display of authority and its impact on community trust.

## Findings

### Introduction of VHTs: misinformation, hope and excitement

The introduction of the VHT strategy in Luwero was welcomed with hope and excitement in the community. Many people gladly anticipated having a group of “doctors” (*abasawo*) in their village to whom they could turn when illness struck them. At first, information about the VHTs was spread through rumour, and the community was ignorant about who these doctors were going to be and how they would be chosen. The process of informing the community about the VHT strategy was poorly managed in terms of clarifying who informs the community, what they tell the community and in how. Community members seemed to have received unclear information about the VHTs and what to expect from them as the information was generally spread informally. Through informal conversations, we learned that some expected that the training of VHTs meant that health services would be closer to their villages. This expectation not only brought high hopes but also some strife and competition among those who wanted to become the “village doctors”. George, a 30-year-old man, expressed this hope in an interview:

The rumors were that we are going to have village doctors. We thought that they are going to have medicine and treat us when we fall sick. We heard that they would be trained to treat diseases, so that we do not need to go to Kasana Health Center every time that we fall sick.

Sunday, a man aged about 40, explained why community members were excited when the idea of VHTs was introduced:

We were told and everybody heard that the selected persons would be the equivalent of medical doctors, where we could go if we fell sick. They were going to be trained to treat malaria and other small illnesses and also be given bicycles to transport the sick to the health center. Everybody was happy that finally the journey to Kasana [where the health center is located] is going to be reduced. The health centers are far and malaria medicines are expensive. That is why everybody was excited that at last the government had remembered to bring services closer to the people at the village level.

It is common that people living in rural areas of developing countries like Uganda have challenges in accessing health services, and sometimes, even when they manage to access the health centre, they find that drugs are out of stock. The promise that VHTs, fellow residents in the community, were to be equipped with essential drugs led to hope and excitement.

### Selection of VHTs: sidelining the community

Membership of a VHT was perceived as influential in the community and so attracted a lot of interest. The guidelines established by the Ministry of Health provided that VHT members must be selected by the community through popular vote. Each VHT was to be composed of about five people, depending on the size of the village, with each team responsible for about 30 households. Political leaders such as village council committee members were not eligible for membership in order to ensure checks and balances.

Despite these guidelines, village council leaders influenced the process and appointed themselves to the VHTs. In all of the villages in the area studied, chairpersons of local councils were members of the VHTs, after being required by the sub-county governments to mobilize and sensitize their respective communities for VHT selection. Local council chairs also selected other members. Concerning her selection as a member of a VHT, Sharon, a 58-year-old woman, said:

It is the local council chairman who knows the way we were selected. They were assigned with the task of looking for people who can read and write. When the chairmen sent the names of the persons they had selected, they informed us when the time for training had arrived. After the training we were allocated homesteads to oversee concerning issues of health.

Nakimuli, a 62-year-old female VHT member, similarly told us:

I was told by the chairman that I was selected to be trained to take care of our community and guide people in health issues. He told me I was selected because I was a friend and a trusted person in the community.

It appeared that the local leaders had usurped the community’s power to vote and select people to the VHTs. This was further confirmed when one evening, while walking through a trading centre past a bar, one member of our research team overheard people asking each other about him, which compelled him to join the conversation. In the discussion that ensued, a person who had participated in an FGD conducted earlier told the group that the researcher was studying the VHTs. They were all eager to find out what the VHTs were doing. One man, about 50 years old, said:

We cannot be sure what they [VHTs] are doing, because the district people came here and gave them money to help the community. As far as we can tell, they are not doing anything. … The chairman selected the names of the people he wanted and sent their names to the district—we heard that they had been called and then we only heard that they are getting money. The chairman and his deputy are the ones who always know what is happening. … These days the money must be finished because they are doing nothing and you do not hear them talking about being VHTs.

From this comment and others like it, we learned that many people in the community thought the VHTs were paid. They did not seem to believe that the VHTs were supposed to work as unpaid volunteers to help fellow community members. The manner in which the local leaders handled the selection seemed to have fueled the suspicions of many in the community who though that VHTs were profiting in the name of helping the community. In another village, a member of our research team was having an informal conversation with Scovia, a 42-year-old woman, who talked about her community’s leaders:

I told you that these people always do things among their own cliques. They will make sure that the rest of the community will not have their way. When Annet [a member of the VHT] left, she was replaced by Sarah. Can anyone honestly say that they are surprised the chairman chose her? Don’t you see that she is in the same group of acquaintances with the chairman? Of course, I cannot say for sure whether that is the reason, but let me also ask you: Why didn’t they choose another one? Those people will be the ones doing everything. I do not think they are bad, but they reflect one side. Why was she chosen to become a VHT for people of the other side, [who] even do not know how she was chosen?

Annet’s replacement, Sarah, also served as the vice chairperson of the village council. During a conversation with the Kagugo parish chairman of the VHTs, we talked about the role of local council leaders deciding the membership of the VHTs instead of the community voting to select those they were comfortable with. He said:

People in our communities are very stubborn and they do not respect authorities anymore. Democracy has blocked their ears from listening. … People are not seeing us as friends and they think we are a burden. When we go to them, they say, “Here they are again, now what do they want?” Before we know it they have turned hostile. They do not want to respect the fact that we have something good for them. If we are to follow what the community wants, we would stop everything and choose new VHTs. If you call them for a meeting they will not come, but will complain if we choose for them. Even if they choose their very best friends, they will not listen to them. The people are lazy on issues of sanitation and hygiene—that’s why they hate us.

This leader dismissed the community’s concern as a non-issue and seemed to place himself and other VHTs apart from the community, which is indeed, we found, a significant problem.

### Literacy requirement: excuse for sidelining community in selection of VHTs?

One of the Ministry of Health-mandated qualifications for those selected to serve on the VHTs was the ability to read and write, at least in a local language. In an interview with the local council leader of Sakabusolo, we were told that this mandate made it easy for him to choose those he knew to be literate. Asked whether that requirement could have influenced his community’s perception of the VHTs, he said:

Local council leaders were invited to the sub-county where they told us that the people to become VHTs should be able to read and write, especially in Luganda. Even then, many of the documents were in English. So, when we came to the community and told them that not everybody was qualified, some people did not believe us. There are not so many people who can read and write in this community. The choice for me was then easy, as I could count them on my finger.

In another conversation with the chairwoman of Kagugo village, we were told how the literacy requirement turned out to work against some of those who could have been VHTs:

In my village there are very few people who know how to write their names. At the sub-county, before they trained us, we had to write our names and parish on a piece of paper. I did not want my parish to be ashamed by sending people who cannot write their name. If someone cannot write their name, but is loved by everyone, you cannot send their name. It was hard to get five literate people to send to the sub-county who were willing to volunteer in this village because they work in other places.

These statements reveal that local leaders found it convenient to make their own choices using the literacy requirement as an excuse. The requirement seemed to constrain the community’s choice of who could serve them as a helper. Informally, some of the community members remained sceptical and suspicious, wondering if local leaders might have invented the requirement in order to influence the selection.

Interestingly, the district health officer for Luwero doubted the capacity of VHTs with basic literacy to manage childhood illnesses collection of health data. In the evaluation report for AMREF’s project, he stated that he preferred that VHTs meet higher educational qualifications, if they were intended to competently manage those roles.

### Community distrust towards selected VHTs: “Those are not the ones who help us!”

There was a sense of distrust and resentment of VHTs because many members of the community felt that the way in which VHTs were selected ignored their preferences. They reasoned that since VHTs were supposed to be community helpers, the community should have had a greater say in their selection than the local council leaders. They became frustrated and suspicious of the government’s and AMREF’s stated intentions to offer help. This frustration was evident when one woman, aged about 40, while in a conversation with other community members, said:

These people [from AMREF] who come to this community pretending to help us should stop lying to us. They are also working for their other goals. … If they want to help us, how could they agree to work with people they know clearly were not chosen by the community? If they come and ally with the chairman and his friends, are they helping us? If they wanted to help us, they should have asked us, because we know the people that can help us. Have they ever seen anybody going to the chairman for help? Only Kyambadde, among the VHTs, helps people, but I think that the rest of them are interested in stealing whatever is sent to the community.

When this respondent articulated both her distrust of the VHTs’ intentions and her suspicion that they were stealing, others listening nodded their heads in agreement. The reputation of the VHTs was clearly tarnished. Lydia, a woman aged about 45, told me of her experience with the VHTs:

The VHT reported me to the sub-county that I did not have a toilet. But since I am not a man and this home is not mine, they were supposed to go to my husband, who had abandoned me. On the VHT, it was Kyambadde who understood my problems since my husband left me with children. She came to my home and we talked and she went to inform the sub-county officials to look for him. She is a very kind woman. She does not hate people and she does not judge without listening to you.

Even when people distrusted and suspected many members of the VHT, they were able to identify others whom they perceived to be good. They appreciated that someone could listen and talk to them and understand their problems.

In an interview, the health assistant responsible for supervising all VHTs in Luwero sub-county stated that she was aware that local council leaders were VHT members in many villages, contrary to the Ministry’s guidelines. However, she seemed to have gone along with the selection of VHTs, saying:

Community members are stubborn and hard to manage. When they are called for meetings, they do not come but show up to complain when you decide for them. AMREF gave us money for sensitization meetings but when community meetings did not happen in the stipulated time, they became impatient. Concerning the issue that some people in the community take all the opportunities,—sometimes it is due to stringent requirements like the ability to co-fund [to contribute some resources]. In most cases it is the leaders who are able.

The assistant appeared reluctant to ensure that the community had their say in the selection of VHTs. She easily sided with the leaders’ version without listening to the other community members. Since she supervised the selection and did not take a keen interest in ensuring that the guidelines were followed, she became an accomplice in elite capture. While the relationship between the VHTs and the community lacked trust and was instead filled with suspicion and misunderstanding, an AMREF report was largely silent on this dynamics save for the recommendation that authorities and their development partners should look for appropriate methods to select motivated volunteers.

### VHT adaptations as a result of resentment from the community

Initially, each VHT member was to be allocated about 25 households, all of which would have participated in selecting him/her. However, we found that in Luwero VHTs instead began working in groups, visiting homes together. Among their first assignments was sensitizing the community on hygiene and sanitation, but many people resisted these efforts and did not welcome or listen to the VHTs. During an FGD with VHTs in Kyetume, one said:

Some of them were very stubborn and not willing to cooperate with us. They would even ask us who made us their boss. They claimed that the government had given us money to construct toilets but instead we were asking the households to do it themselves!

A similar scenario was mentioned in an FGD with VHTs in Sakabusolo, when another VHT member described their challenges:

We went somewhere and then a man wanted to cut us with machete. He was arguing that he was poor and we [had] come to tell him useless things. He said that if we want them to have a toilet, we should build it. He claimed that we are being paid a lot of money. One day I tried to explain that we volunteer but no one believed [me]. They demanded that we share the money with them.

In an interview, Tito, a VHT member, said that VHTs believed that they might have more success in groups because community members might know at least one member of the VHT:

We decided to go in groups to avoid those questions from the community. They will surely not say “who are you?” when she comes with other VHTs whom they know.

When asked if it would not be simpler to do a one-on-one visit between a VHT and someone from each of the households, as that would be much friendlier than a group of five people coming in at once, Tito replied:

People in this community are hard and they do not want to be advised. It is when things are too hard for them that they become humble. So we decided to go in groups to make it hard for them to attack us as they did when people tried to go as individuals.

The VHTs began working as groups because they did not get a friendly reception. The adaptation meant they had to walk longer distances as a group, to cover all the homesteads, rather than each walking only to the homesteads allocated to him/her. This later played a role in their loss of morale for their work.

### VHTs as friendly visitors or as sanitation inspectors: the dilemma

There is one time I did not go to the field—my colleagues told me that some people in Bukuma village ran away when they saw the team approaching their homes because they did not have latrines.

The above quote from Sajjabi, a 58-year-old woman and VHT member, illustrates the problematic relationship between the VHTs and some members of the community. The friendly team of helpers sometimes created fear among the community members due to the power and authority they used to enforce their work. In an FGD, exchanges between VHTs and other community members illustrated the how the work of the VHTs proceeded from providing sanitation advice to a campaign of sanitation inspections:

VHT-1: It was not easy to convince someone that the toilet is in their own interest. Sometimes the people became harsh though some later accepted our advice. But sometimes we could be forced to arrest those who don’t see what we were telling them to be useful.

Interviewer: How did you arrest them without the police?

VHT-1:We could take the report to the sub-county, of all those people who refused to have toilets. Then the sub-county offices would send soldiers to arrest them and we would give clear directions to the homesteads.

R4: But in your method of work, I don’t think you just go abruptly and arrest him. You first go to him, warn him and educate him about the benefits of having a latrine/toilet. You only arrest him when he refuses.

VHT-2: But if he fails to listen to me and I report him, they begin complaining that we are harsh. For example at Bwaziba, VHTs there invited us to arrest some family without toilets because they feared to arrest them and then be hated in their own village. So we went and did the work for them.

The VHTs found themselves in a dilemma: they were working as inspectors and using a force that did not portray them as helpers. As they did not like to be viewed like that in their own communities, they opted to swap villages with their colleagues from neighbouring villages. The chairman of the VHTs in the parish told me:

People need an iron hand because they do not listen. But the last time we inspected homesteads, some people were harsh, which intimidated many of our colleagues and we became demoralized. Sometimes the language that the people will hear is that the one that scares them.

The use of the words “arrest”, “hate” and “iron hand” in these conversations show that the relationship between VHTs and the community had deteriorated. The VHTs found themselves having to issue threats of arrest for non-compliance with sanitation rules. This kind of relationship demoralized the VHTs themselves who did not want to create grievances with community members.

## Discussion

Though the use of volunteer community health workers has become popular as a means of achieving health goals in communities in the developing world, there is little consensus on how to address the challenges of attrition that have plagued many CHW programmes [59-61]. The need for creativity in finding ways of sustaining CHWs remains urgent [25]. Communities are heterogeneous and require tailored approaches and flexibility to strengthen CHW programmes. The natural helper model was developed to provide this kind of flexibility. Its main assumption is that in any community there exist persons to whom others turn for help as a result of the mutual trust and reciprocal support mechanisms inherent to that society. It is therefore suggested that community programmes seeking to use volunteer lay health advisers would benefit by identifying these natural helpers and recruiting them to serve more formally in their own communities [39].

As early as 1996, it was concluded that the effectiveness of volunteer CHW projects depends to a large degree on the people involved. Evaluations of CHW programmes, however, have not shown how they measure the extent to which CHWs are representative of the communities they are selected from. Thus, it has always been assumed that CHWs represent the community since they reside there [25]. This case of VHTs in Luwero demonstrates that this assumption may not always be correct.

Ethnographic research can provide insights into community-level processes that are usually missed by surveys and other methodologies. Our findings show what may happen when the natural helper model is not used in the selection of CHW volunteers. Firstly, stating that the community would choose the members of the VHTs may have created false expectations. As it happened in this case, the community was manipulated through misinformation and half-truths in a systematic process that saw the power of the community to select their own representatives usurped by their local leaders. This power grab may have been an unintentional result of a badly executed process, especially since the supervisors did not ensure that local village leaders followed the guidelines. Though local leaders are chosen by the people and therefore may have a mandate, it should be noted that the manner of selecting village leaders in a political process draws upon different interests than the process of selecting community volunteers. It has been argued that different constellations of interests make up the political landscape of a community [62,63]. In this paper, we see how those who took charge of selecting members of the VHTs ignored the concerns raised by community members. The local government and AMREF staff, who were supposed to supervise the whole process, did not interest themselves in how the selection of VHTs was carried out on the ground to ensure that the guidelines were followed by the local authorities, thus ignoring the evidence, documented in the community development literature, that local leaders act as “gate keepers” to their community and may usurp the power of the local communities as shown in typical cases of elite capture [64,65].

Secondly, guidelines set by the national government without input from the local community will not reflect local realities. Lack of flexibility in the guidelines to suit each community opens up the way for manipulation that even proper supervision may not help. For instance, setting literacy requirements for VHTs played into the hands of powerful local leaders who are usually among the few literate people in a typical rural community. Not enforcing the prohibition on local leaders serving on VHTs also allowed the local leaders to usurp power. Both laxity and strict adherence to the guidelines served the interests of those already in powerful positions in the community.

Thirdly, our findings show that the majority of the members of the community did not consider the selected VHTs to possess the characteristics of helpers. This means that communities have their own expectations about who is best positioned to help. Indeed, many people in the community were frustrated that they were not consulted, as they felt they had better knowledge of the people they trust to help them. This means that in initiating the concept of VHTs, the government may not have fully understood that communities have their own helping structures that they trust and support.

Lastly, because the majority in the community did not trust the helping credentials of those selected to help them with health-related issues, they resented them and denied them the expected community support. This is what happens when communities are sidelined in issues that pertain to them, when they feel they ought to be consulted and their opinions taken seriously [65]. To adapt to this resentment, the VHTs altered their working methods in ways that sought to use authority and power that was not derived from the community mandate and thus alienated them further. Because many on the VHTs were local leaders, once they lost community support, they resorted to relying on the authority of their leadership positions. This is perhaps why the Ministry’s guidelines explicitly barred local leaders from being members of the VHTs. The VHTs, who were supposed to be trusted and friendly helpers, soon turned into a force that was feared and resented by those whom they were meant to help. In the absence of financial incentives, the resulting scenario was ripe for a decline in motivation among VHTs and their eventual resignation.

The natural helper model advises that identifying the “right persons” from informal helping networks should precede the determination of training and work requirements; such requirements should fit those selected [39,41]. Accordingly, the chosen individuals in that group would be highly respected, trusted in the community and more likely to be motivated to volunteer. The NHM offers a participatory process that locates the selection of community volunteers at the grass-root level in communities and may avoid the setbacks resulting from elite capture, as documented by the findings described in this paper. The natural helper model offers a framework through which the community would be more engaged in order to select VHT members that are trusted and supported by the community members and lead to better outcomes in CHW programmes.

### Limitations of the study

This qualitative study was carried out in one rural community in central Uganda, and while the results provide important insights into the factors that play a role in the functioning of village health teams and contribute to the understanding of processes of volunteering, recruitment and motivation more generally, they remain limited in their generalizability. Having said that, the study community is fairly typical of many rural communities in Africa, and consequently, results are likely to be transferable to other similar settings in Uganda and perhaps beyond.

## Conclusion

The NHM process better identifies the people who will freely and informally interact with the target community without appearing confrontational or exhibiting unnecessary power and authority. As has been noted, the value of the community health worker is in his/her embeddedness in the community [11]. The NHM offers a framework for identifying those natural helpers already embedded in the community by surveying the informal helping networks. The actual process of identifying these informal networks and discovering the various “natural helpers” to whom nodes of community members are linked may be a time-consuming process. However, for long-term national programmes for whom sustainability is key, the trade-offs between time spent and quick recruitment is worth it. If tried with due diligence and given the time and resources necessary for initial exploration, the NHM thus may offer a framework for the selection of volunteers who have the trust and support of community members, thereby allowing health and other social service information to be disseminated.

## Abbreviations

AMREF: African Medical Research Fund
CHW(s): Community health worker(s)
NHM: Natural helper model
PHC: Primary health care
VHT(s): Village health team(s)
